# Exploring the Neuroprotective and Neuropsychiatric Symptom Management Potential of Ketamine in Alzheimer’s Disease

**DOI:** 10.7759/cureus.86855

**Published:** 2025-06-27

**Authors:** Amy Avakian, Zachary I Merhavy

**Affiliations:** 1 Department of Diagnostic Radiology, Ross University School of Medicine, Pontiac, USA; 2 Department of Clinical Medicine, Ross University School of Medicine, Pontiac, USA

**Keywords:** alzheimer’s dementia, alzheimer's disease, anesthesia, ketamine, ketamine alzheimer's, neuropharmacology, nmda receptor antagonist

## Abstract

Alzheimer’s disease (AD) is a progressive neurodegenerative disorder characterized by cognitive decline, synaptic dysfunction, and neuroinflammation. Despite extensive research, current therapeutic options offer limited symptomatic relief without altering disease progression. Ketamine, an N-methyl-D-aspartate receptor (NMDAR) antagonist traditionally used as an anesthetic, has garnered attention for its rapid-acting antidepressant effects and potential neuroprotective properties. This narrative review examines ketamine's emerging role in AD, focusing on its mechanisms of action, including modulation of glutamatergic transmission, enhancement of synaptic plasticity via brain-derived neurotrophic factor (BDNF) pathways, and attenuation of neuroinflammatory processes. This paper also explores ketamine's efficacy in managing neuropsychiatric symptoms prevalent in AD, such as depression and agitation. While preliminary findings are promising, further research is necessary to establish ketamine's safety and efficacy in this context.

## Introduction and background

Alzheimer’s disease (AD) affects over 55 million individuals worldwide, posing significant challenges not only to healthcare systems but also to the informal caregivers who provide the majority of long-term support [1]. Compared to other caregiving populations, caregivers of patients with Alzheimer’s and related dementias report higher levels of psychological stress, depression, and physical strain. Many provide round-the-clock care for extended periods, often sacrificing their own health, social life, and financial stability [1]. These caregivers describe the experience as a “mantle of responsibility” marked by grief, loneliness, and a loss of agency, underscoring the bidirectional toll of Alzheimer’s on both patients and their families [1].

Current pharmacological treatments, such as cholinesterase inhibitors and memantine, provide modest symptomatic benefits without halting disease progression. The glutamatergic system, particularly N-methyl-D-aspartate receptor (NMDAR)-mediated excitotoxicity, plays a pivotal role in AD pathophysiology. Ketamine, a non-competitive NMDAR antagonist, has demonstrated rapid antidepressant effects and potential neuroprotective mechanisms, making it a candidate for repurposing in AD treatment strategies [2,3].

## Review

Pathophysiology of AD

**Figure 1:**
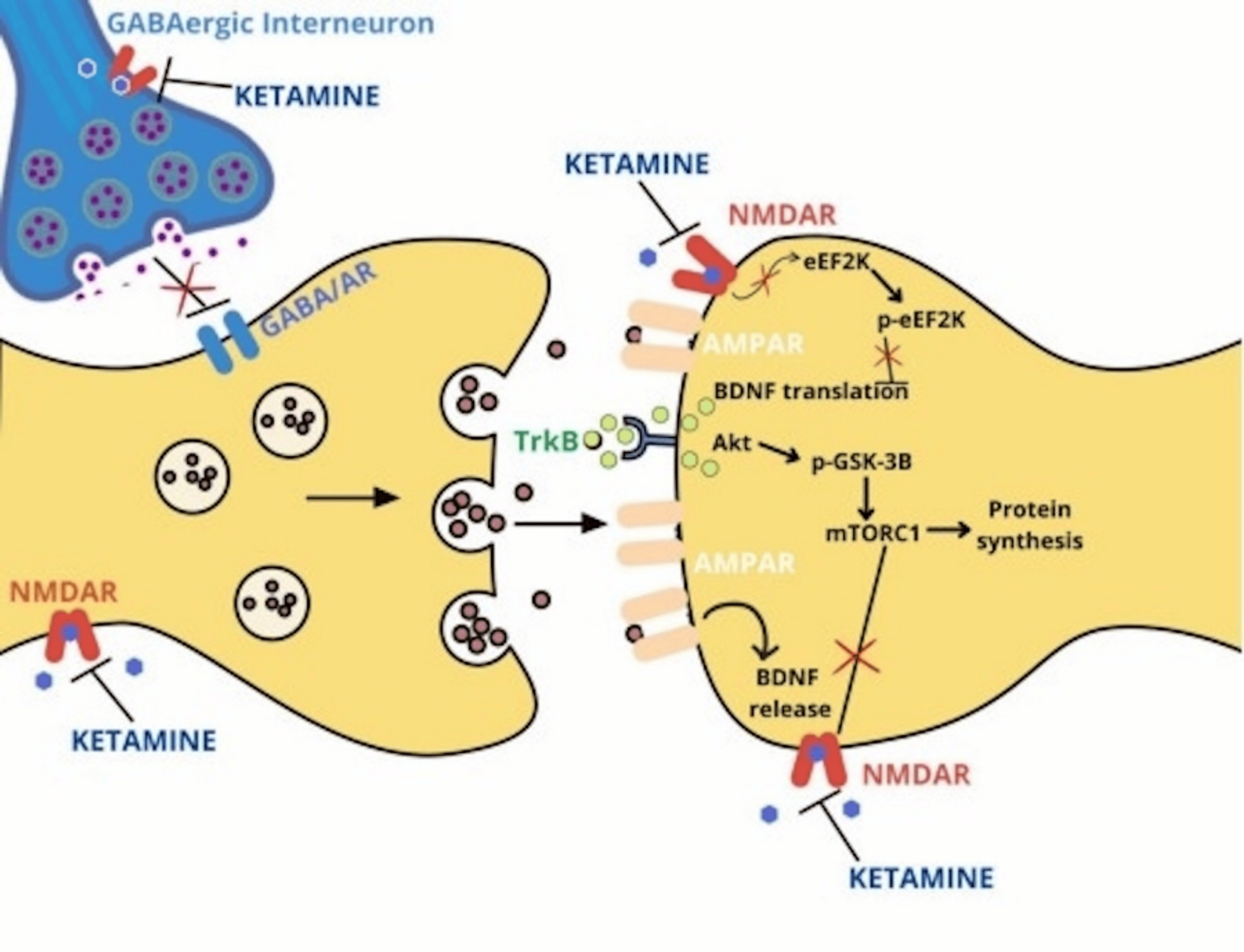
Mechanisms of Action of Ketamine Source: reference [6] (Creative Commons Attribution-NonCommercial-NoDerivatives 4.0 International (CC BY-NC-ND 4.0))

**Figure 2:**
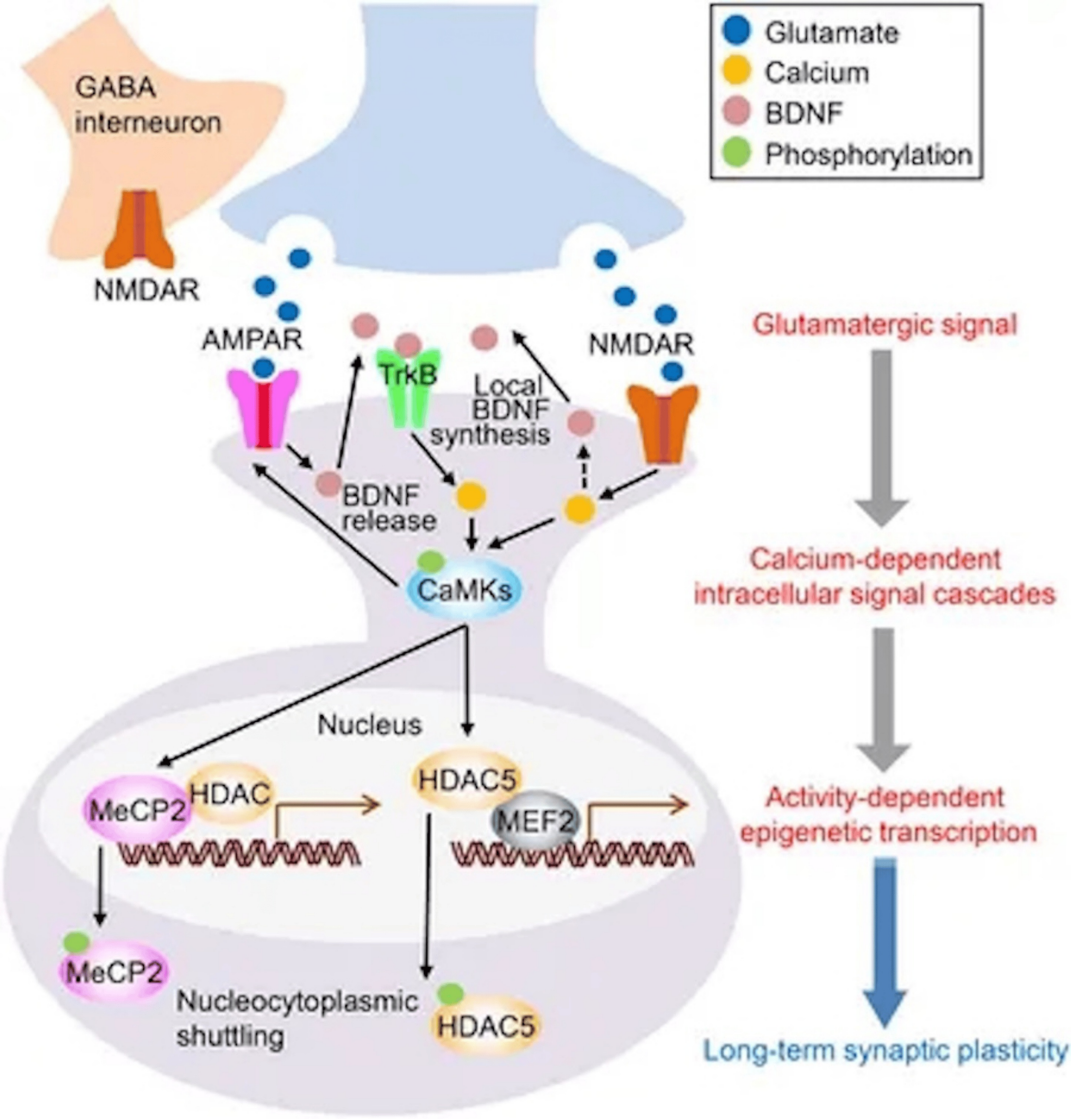
Proposed Mechanisms of Ketamine's Antidepressant Actions Source: reference [7] (Creative Commons Attribution 4.0 International (CC BY 4.0))

AD is marked by the accumulation of amyloid-beta (Aβ) plaques and neurofibrillary tangles composed of hyperphosphorylated tau protein, leading to synaptic loss and neuronal death [2]. Glutamate-induced excitotoxicity contributes to synaptic dysfunction, while chronic neuroinflammation exacerbates neuronal damage [2,4]. Neuropsychiatric symptoms, including depression, agitation, and anxiety, are common in AD and significantly impact patient quality of life and caregiver burden [5]. Neuroinflammation is not merely a secondary reaction to neuronal degeneration, but a driving force of pathology in AD, contributing as much or more to the pathogenesis as do the plaques and tangles themselves [4]. This reframes inflammation not as a byproduct but as a core pathologic agent (Figures 1, 2) [4].

Ketamine: mechanism of action and historical use

Ketamine exerts its effects primarily through NMDAR antagonism, reducing glutamatergic excitotoxicity [8]. Additionally, ketamine influences synaptic plasticity by enhancing BDNF expression and activating the mammalian target of rapamycin (mTOR) signaling pathway, promoting neurogenesis and synaptogenesis [3,9]. Activation of the mTOR pathway is noteworthy since mTOR is a central controller of protein synthesis required for new synaptic connections [2]. These mechanisms suggest potential therapeutic benefits in neurodegenerative conditions like AD [10]. Emerging evidence suggests that the antidepressant effects of ketamine are mediated not only by NMDAR blockade but also through enhanced alpha-amino-3-hydroxy-5-methyl-4-isoxazolepropionic acid (AMPA) receptor throughput, which increases synaptic excitation and initiates downstream neurotrophic cascades [3].

The activation of AMPA receptors appears to be necessary for the behavioral and biochemical effects of ketamine, suggesting a shift in focus from NMDAR inhibition to glutamate-mediated synaptic potentiation [3]. Ketamine is the prototype for a new generation of glutamate-based antidepressants that rapidly alleviate depression within hours of treatment [11]. This rapid action is attributed to ketamine's ability to enhance synaptic connectivity and function, offering a new paradigm in antidepressant therapy [11].

Ketamine’s neuroprotective potential

Preclinical Evidence

In animal models, ketamine has demonstrated neuroprotective effects by mitigating neuroinflammation and oxidative stress as well as by enhancing cognitive function [10]. Documented effects of ketamine on inflammatory cytokines add to the rationale for its study in AD [2]. Misfolded and aggregated proteins bind to pattern recognition receptors on microglia and astroglia and trigger an innate immune response, which induces a cascade of inflammatory mediators that contribute to disease progression and severity [4]. This emphasizes the relevance of microglial suppression and cytokine regulation and positions microglial regulation as a viable therapeutic target in AD - one that ketamine’s pharmacology may intersect with directly [4]. Ketamine reduces the production of pro-inflammatory cytokines such as TNF-α and IL-6, also decreasing microglial activation, making it a potential candidate for conditions marked by neuroinflammation [10]. Given that microglial overactivation is central to the neurodegenerative cascade in AD, this aligns with ketamine’s putative therapeutic value in modulating the immune landscape of the AD brain [10].

The ketamine metabolite (2R,6R)-hydroxynorketamine (HNK) has been shown to rescue hippocampal synaptic plasticity and memory deficits in AD mouse models [12]. Interestingly, (2R,6R)-HNK does not appear to inhibit NMDARs at therapeutically relevant concentrations, yet it still produces sustained antidepressant-like effects on rodent models [3]. This suggests that some of ketamine’s therapeutic benefits may arise independently of its dissociative and psychotomimetic properties [3].

Beyond synaptic restoration, HNK has been shown to activate key signaling cascades implicated in memory formation and neural repair [12]. Specifically, HNK promotes ERK1/2, mTOR, and p70S6K/RPS6 signaling pathways - all of which regulate mRNA translation, protein synthesis, and synaptogenesis in hippocampal neurons [12]. These cascades are notably disrupted in AD, and their reactivation is associated with improved plasticity and learning capacity [12].

Moreover, HNK corrected aberrant transcriptional signatures related to inflammation, innate immunity, and proteostasis in aged APP/PS1 mouse models of AD [12]. This indicates that its therapeutic potential may extend beyond synaptic modulation to include broad transcriptional reprogramming of disease-relevant networks [12]. These findings highlight (2R,6R)-HNK as a mechanistically distinct and promising candidate for future AD therapies [12].

A subanesthetic dose of ketamine is a powerful activator of multiple parallel neurotrophic signaling cascades with neuroprotective actions that are not always NMDAR-dependent [10]. This reinforces the idea that ketamine may engage alternative intracellular mechanisms relevant to AD pathology and that its benefits could be decoupled from its acute psychoactive profile [10]. Ketamine has the potential to affect several aspects of dementia care by targeting multiple hypothesized neurobiological mechanisms that lead to behavioral disturbances, suggesting its multifaceted role in addressing AD pathology [5].

Clinical Observations

One recent case detailed the use of intravenous ketamine in a 48-year-old patient with early-onset dementia, resulting in rapid improvements in mood, cognition, and daily functioning [9]. While anecdotal, such findings underscore the need for controlled clinical trials to evaluate ketamine's efficacy in AD patients [9].

In a notable case, a 56-year-old female patient with early-onset dementia exhibited significant cognitive improvements following a series of intravenous ketamine infusions over two months [9]. The patient reported enhanced mental clarity, increased focus, improved memory, and elevated energy levels [9]. Quantitative assessments revealed substantial gains in that the neurocognitive index improved from the first to the 19th percentile, composite memory from the 16th to the 37th percentile, visual memory from the 10th to the 42nd percentile, executive function from the first to the 34th percentile, and reaction time from the fourth to the 16th percentile [9]. Ketamine has been found to have a significant effect on inflammation by blocking cytokine production and inflammatory cell recruitment, thus leading to a profound and systemic anti-inflammatory response [9]. These findings suggest that ketamine may exert neuroprotective effects in dementia, potentially through its anti-inflammatory properties and modulation of NMDAR activity [9].

Managing neuropsychiatric symptoms in AD

Depression and agitation are widely prevalent in AD, often leading to increased morbidity and caregiver stress [1]. Traditional treatments, such as selective serotonin reuptake inhibitors (SSRIs) and antipsychotics, have limited efficacy and potential adverse effects in this population [1]. Ketamine's rapid antidepressant properties and potential to alleviate agitation offer a promising alternative [5]. However, evidence remains limited, and further studies are warranted to assess its safety and effectiveness in elderly and cognitively impaired individuals [5,10]. The use of ketamine in distinct clinical scenarios including depression with catatonia, agitation, and treatment-resistant depression emphasizes the versatility of ketamine in managing diverse neuropsychiatric symptoms in AD [5].

Over the past decade, there has been replicated evidence demonstrating the rapid and potent antidepressant effects of ketamine in treatment-resistant depression [10]. This underscores the potential of ketamine as a valuable tool in addressing the complex neuropsychiatric manifestations of AD [11].

Ketamine has additionally been cited as an exciting alternative to electroshock therapy in treatment-resistant depression, especially in patients with a high risk of suicide, suggesting its potential utility in more widely managing severe neuropsychiatric symptoms [2].

One study found that patients experienced not only cognitive enhancement following ketamine administration but also notable improvements in mood and daily functioning [9]. The report stated that after just the first round of infusions, patients reported decreased depression, reduced brain fog, and increased mental clarity [9]. These benefits facilitated a reduction in one patient’s quetiapine dosage and discontinuation of rivastigmine, further indicating ketamine's potential in alleviating neuropsychiatric symptoms associated with dementia [9].

Risks, limitations, and ethical considerations

Clinical and Safety Limitations

Regardless of the countless potential benefits of ketamine use in these settings, ketamine's psychotomimetic effects and potential for abuse have raised concerns, particularly in vulnerable populations like AD patients [5]. Long-term safety data are inadequate and sparse, and ethical considerations regarding informed consent and risk-benefit analysis are paramount in this demographic [5]. Until more substantial evidence for the use of ketamine among older adults with dementia is available, case-based evidence can only serve as a preliminary, albeit relevant, segment of the literature supporting its use among these individuals [10]. The high potential of ketamine’s use in these case studies drastically underscores the need for rigorous clinical trials to establish safety and efficacy [5]. Other factors that have hindered further study with ketamine have been cited as the potential short-term side effects profile, the stigma surrounding the drug’s use, and cost [9].

Regulatory hurdles and the need for rigorous clinical trials must be addressed to advance ketamine's application in AD treatment [2,11]. The risk/benefit considerations for chronically treating AD patients are quite different than those for acutely preventing suicide in severely depressed patients, further underscoring the need for careful evaluation [2]. Notably, the side effect profile of ketamine is dose-dependent, and its metabolites such as (2R,6R)-HNK may offer therapeutic advantages without inducing dissociative symptoms or abuse potential [3]. Given the long disease course of AD and the chronic activation of glial cells, even low-level inflammation can exert damaging effects over decades, raising concerns about the timing and safety of long-term immunomodulatory strategies [4].

Despite its benefits, at anesthetic doses applied during neurodevelopmental windows, ketamine contributes to inflammation, autophagy, and apoptosis and enhances levels of reactive oxygen species [10]. This duality highlights the importance of dosing, timing, and patient selection when considering ketamine in vulnerable populations such as older adults with AD [10]. Thus, while ketamine’s anti-inflammatory and neurotrophic effects support its clinical exploration in AD, its potential neurotoxicity under certain conditions requires stringent safety protocols and long-term surveillance in trial designs [10].

Ethical and Systemic Barriers

While the therapeutic outcomes are promising, further challenges related to the accessibility and affordability of ketamine treatment play another major role in the blunted momentum of ketamine’s widespread use [9]. One case highlighted that a patient who had been successfully treated with ketamine eventually discontinued therapy due to financial constraints surrounding the cost of treatment [9]. This only further emphasizes how the need for broader insurance coverage and cost-effective treatment protocols play considerable roles when treating these patients [9].

## Conclusions

As the burden of AD continues to escalate, the search for innovative, effective, and scalable treatment strategies becomes increasingly urgent. Existing pharmacologic therapies offer limited symptomatic relief and do not alter the underlying course of the disease. Ketamine, traditionally used as an anesthetic and more recently as a rapid-acting antidepressant, has emerged as a potential candidate to fill this therapeutic gap. Its pharmacological profile includes modulation of glutamatergic transmission, attenuation of neuroinflammation, and promotion of synaptic plasticity, all of which align with key pathological mechanisms implicated in AD. Beyond its neuroprotective effects, ketamine also holds promise in addressing the often-overlooked neuropsychiatric symptoms of Alzheimer’s, such as agitation, depression, and anxiety. These symptoms significantly impair quality of life for patients and impose emotional and physical strain on caregivers. Addressing both the biological and behavioral dimensions of AD is essential for improving comprehensive care outcomes.

However, despite these promising attributes, ketamine’s use in this context remains largely investigational. There are critical gaps in knowledge regarding its long-term safety profile, optimal dosing regimens, tolerability in older adults with cognitive impairment, and potential for sustained clinical benefit. Additionally, ethical considerations surrounding consent and risk-benefit analysis must be carefully addressed, particularly in vulnerable populations. Future research should prioritize rigorous randomized controlled trials that evaluate ketamine’s impact on both cognitive decline and neuropsychiatric symptomatology in diverse patient populations. Multimodal study designs that incorporate imaging, biomarker analysis, and behavioral assessment may offer valuable insights into its mechanisms of action and therapeutic potential. In summary, ketamine may represent a paradigm-shifting intervention for AD, offering a multifaceted approach that targets both the neurobiological and behavioral complexities of this condition. Continued exploration of its clinical applications may open new avenues of hope for patients, caregivers, and clinicians confronting one of the most challenging neurodegenerative diseases of our time.
